# Unusual Magnetic Features in Two-Dimensional Fe_5_GeTe_2_ Induced by Structural Reconstructions

**DOI:** 10.1021/acs.jpclett.2c00692

**Published:** 2022-05-26

**Authors:** Soheil Ershadrad, Sukanya Ghosh, Duo Wang, Yaroslav Kvashnin, Biplab Sanyal

**Affiliations:** Department of Physics and Astronomy, Uppsala University, Box-516, 75120 Uppsala, Sweden

## Abstract

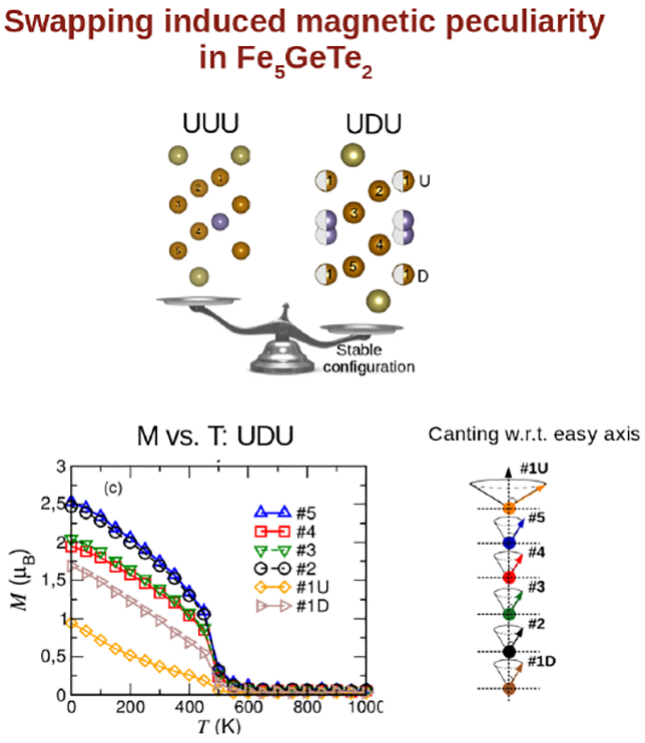

Recent experiments on Fe_5_GeTe_2_ suggested the presence of a symmetry breaking of its conventional crystal structure. Here, using density functional theory calculations, we elucidate that the stabilization of the (√3 × √3)*R*30° supercell structure is caused by the swapping of Fe atoms occurring in the monolayer limit. The swapping to the vicinity of Te atoms is facilitated by the spontaneous occurrence of Fe vacancy and its low diffusion barrier. Our calculated magnetic exchange parameters show the simultaneous presence of ferromagnetic and antiferromagnetic exchange among a particular type of Fe atom. The Fe sublattice projected magnetization obtained from Monte Carlo simulations clearly demonstrates an exotic temperature-dependent behavior of this Fe type along with a large canting angle at *T* = 0 K, indicating the presence of a complex noncollinear magnetic order. We propose that the low-temperature crystal structure results from the swapping between two sublattices of Fe, giving rise to peculiar magnetization obtained in experiments.

On the basis of the Mermin-Wagner theorem,^1^ two-dimensional (2D) materials cannot retain a long-range magnetic order at a finite temperature due to thermal spin fluctuations within the description of an isotropic spin Hamiltonian. In the presence of magnetic anisotropy, however, this restriction is no longer valid, which opens the path for the realization of 2D magnets. Prior to 2016, defect engineering was the main root to induce magnetism in 2D materials.^2^ However, successful exfoliation of 2D Cr_2_Ge_2_Te_6_^3^ and CrI_3_^4^ from their bulk van der Waals (vdW) crystals has opened new doors to the fabrication of stand-alone 2D magnets and their exploitation in next-generation devices. However, the low magnetic ordering temperature in Cr_2_Ge_2_Te_6_ and CrI_3_ with *T*_C_ ≈ 60 K can restrict their functionality. With the advent of metallic vdW magnets, an enormous playground has evolved to study the nature of itinerant magnetic order in 2D materials. Among this family, Fe_*n*_GeTe_2_ compounds possess the highest magnetic ordering temperatures. Moreover, they offer exotic properties suitable for topology-based spintronic applications, such as an extremely large anomalous Hall effect^5^ and the formation of Neel-type skyrmions at room temperature.^6^ However, because of the complexity of structure, the physics behind the exotic magnetic behavior of these materials is not well-understood.^7^ In these crystals, a metallic film of Fe_*n*_Ge is sandwiched between two layers of Te, separating them by a vdW gap. Ferromagnetically ordered Fe_3–*x*_GeTe_2_^8^ shows *T*_C_ at ∼230 and 130 K, in bulk and monolayer forms, respectively. It was shown that, in few-layer flakes of Fe_3–*x*_GeTe_2_, *T*_C_ can increase over 300 K by electrochemical gating.^9^ Importantly, as the thickness of the metallic film increases by adding extra layers of Fe, the magnetic ordering temperature escalates. In bulk Fe_5–*x*_GeTe_2_,^10−12^*T*_C_ varies over a range of 270–310 K, depending on the structural properties and thermal history of grown crystals. Moreover, this temperature can be further enhanced, and a ferromagnetic (FM) to antiferromagnetic (AFM) transition can be induced via transition-metal doping.^13,14^ Furthermore, the magnetic anisotropy energy (MAE) is sensitive to crystal preparation methods, evident in various experiments where out-of-plane and in-plane easy-axis anisotropy has been reported.^10,15^ Because of these complications, a unified picture to describe the temperature dependence of magnetization remains a big missing piece of this puzzle. A detailed theoretical study on Fe_5_GeTe_2_ using an ab initio approach is still less unexplored as compared to other members in the Fe_*n*_GeTe_2_ (*n* = 3,4) family^16−19^ and other vdW magnets like CrX_3_,^20−26^ Cr_2_GeTe_6_,^27,28^ etc.

From the above discussion, it is clear that an intricate relationship between structural, chemical, and magnetic order exists, which should be thoroughly understood. In this paper, using first-principle approaches, we aim to put forward a model that can link between the controversial experimental results and create a unified model to justify the magnetic behavior of this compound in the 2D regime. The weak interlayer coupling found in Fe_5_GeTe_2_ due to its vdW nature is a piece of evidence that indicates a better understanding of the behavior of free-standing monolayer should be the starting point of unraveling the exotic behavior found in the bulk form of Fe_5_GeTe_2_.

Density functional theory (DFT)-based calculations were performed using Vienna Ab Initio Simulation Package (VASP), treating the generalized gradient approximation (GGA) as the exchange-correlation functional.^29^ Unlike some other studies,^30^ we refrained from using the DFT+Hubbard U method, because it produces incorrect ground-state properties, for example, lattice parameter and magnetic moments, whereas with GGA, we reproduce these properties correctly. Moreover, the d-band widths are larger compared to U values, and hence, the use of DFT+U may not be appropriate to capture accurate electronic description and magnetic behavior. The interatomic isotropic symmetric exchange parameters *J*_*ij*_ of the Heisenberg model were calculated using full-potential linear muffin-tin orbital (FP-LMTO) code RSPt.^31^ Monte Carlo simulations using the parametrized Heisenberg model with isotropic and anisotropic terms were performed by UppASD code^32^ to calculate magnetic ordering temperature *T*_C_. Details of the computations are provided in the Supporting Information.

Fe_5_GeTe_2_ monolayer has five atomic layers containing Fe. The Te atoms are situated in two outermost layers, while the Ge layer is placed midway between the Fe layers. Recent experimental studies have reported, among these five Fe atoms, Fe1 situated in the outermost plane of an Fe_5_Ge subunit can be located either directly above or below Ge giving rise to two possible sites for Fe1. This partial occupancy of Fe1 species is revealed in X-ray diffraction data.^10,13,15,33^ Swapping of Fe1 between these two equally probable sites causes splitting of the Ge site as well; that is, when Fe1 is situated above Ge then it pushes down the Ge atom along the *c*-axis and vice versa. Therefore, the splitting of the Ge site is a consequence of Fe1 split sites, confirmed by scanning tunneling electron microscopy (STEM) experiments.^10,15,34^ More interestingly, the scanning tunneling microscopy (STM) topography obtained at the surface of Fe_5_GeTe_2_ has revealed the existence of 3^1/2^ × 3^1/2^ superstructures, which are attributed to the ordering of the Fe1 layer.^33,35^ The intensity of √3 × √3*R*30° reflections is more prominent if a crystal is rapidly quenched.^10^

Motivated by the experimental scenario, we thoroughly investigated the swapped configuration of Fe1 in a √3 × √3 superstructure in which a maximum of three Fe atoms can be placed at a given *c*-plane. Figure 1a shows the splitting of Fe1–Ge pairs where the distance between Fe1 and Ge always remains 2.46 Å (irrespective of either Fe1 is above or below Ge). Split sites of Fe1 and Ge are separated by 5.89 and 0.64 Å along an out-of-plane direction. Therefore, depending on the site occupied by Fe1, Ge is displaced along the *c*-axis to maintain the proper bond distance.

**Figure 1:**
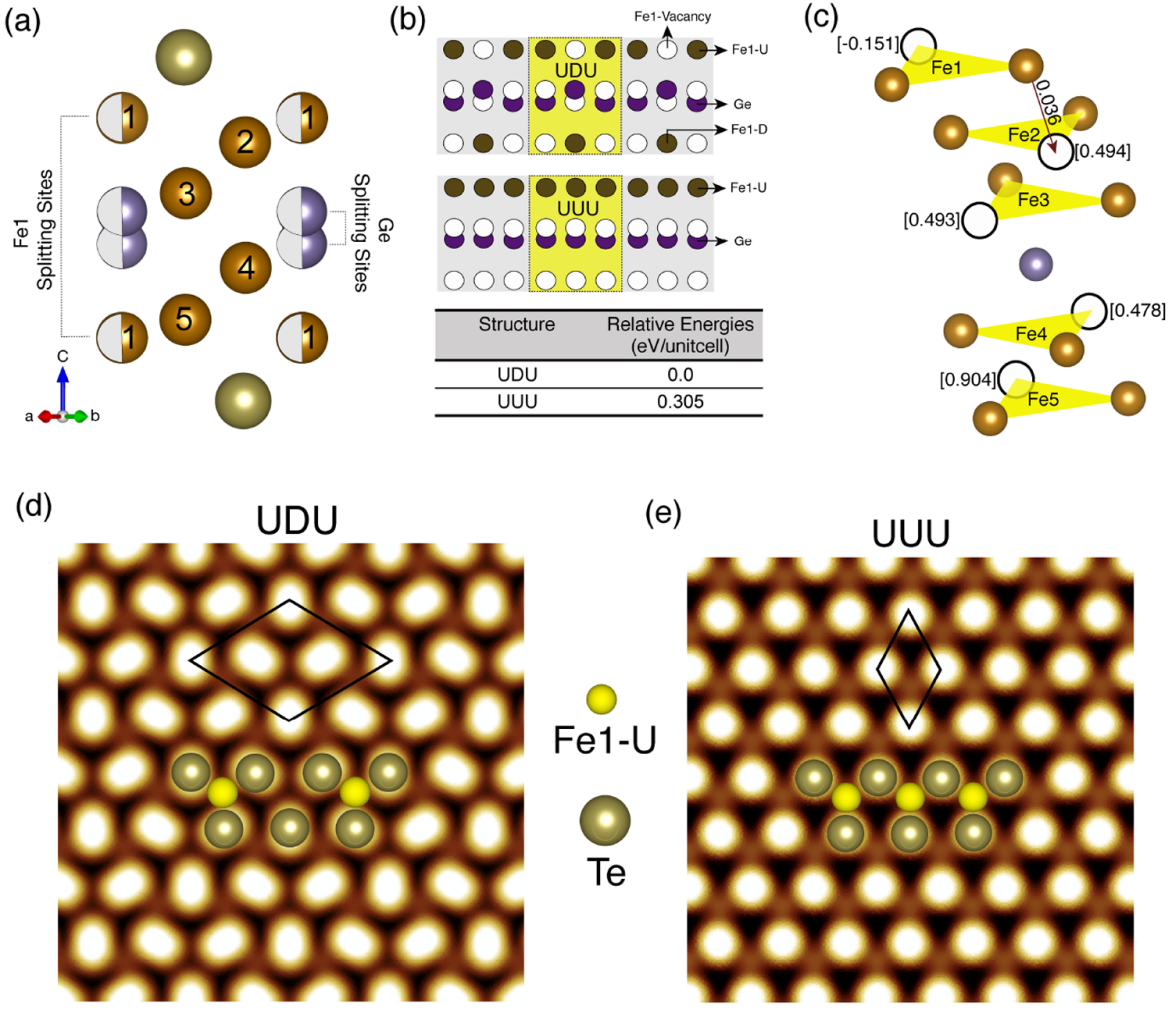
(a) Side view of Fe_5_GeTe_2_ monolayer, with Fe1–Ge split sites. Brown, purple, and dark green spheres show Fe, Ge, and Te atoms, respectively. (b) Schematic representation of UDU and UUU configurations in upper and lower panels, respectively. Circles filled with color and white show the presence and absence of atoms, respectively. (c) Schematic view of Fe vacancy sites with corresponding formation energies and vacancy-mediated diffusion path of Fe atoms results in swapping of Fe1 from directly above to below Ge. Simulated STM images for (d) UDU and (e) UUU configurations, obtained at *V*_bias_ = −0.05 eV, in constant height mode. The brightest spots correspond to the position of Te atoms, whereas semibright spots located at the center of “Y”-shaped features show the positions of Fe1 or (Fe1U) situated directly above Ge, while dark spots occur due to the absence of Fe1 above Ge (see text).

The Fe1–Ge subunit can occupy one of the two possible split sites, that is, either above or below Ge giving rise to two Fe1 sublattices. In one scenario, the Fe1 atoms would be arranged in Fe1 (up)-Fe1 (down)-Fe1 (up) or UDU order; the other energetically degenerate pattern would be Fe1 (down)-Fe1 (up)- Fe1 (down) or DUD. In Figure 1b the upper panel shows Fe_5_GeTe_2_ in the UDU configuration when two of the Fe1 atoms are above Ge, and one Fe1 is situated below Ge in a √3 × √3 cell of the monolayer; filled and empty circles show the presence and absence of any atom, respectively. Fe1–Ge split sites make the crystal structure of Fe_5_GeTe_2_ more symmetric in nature, which contains three inequivalent Fe sites per unit cell: (1) Fe1U and Fe1D, (2) Fe2 and Fe5, and (3) Fe3 and Fe4. Fe_5_GeTe_2_ monolayer can form a lower-symmetry structure where split sites of Fe1 and Ge are absent. According to the recent experimental study by May et al., the higher-symmetry structure is generated from crystals that are vapor grown and quenched from a growth temperature of 1023 K into an ice–water bath, while the lower-symmetry structure comes from crystals that are cooled naturally in the furnace.^12^

Similar to other Fe_*n*_GeTe_2_ systems, experiments on Fe_5–*x*_GeTe_2_ have revealed the presence of Fe deficiency, where *x* varies from 0.04^15^ to 0.28, where Fe1 has the maximum tendency to host a vacancy.^10,13,33,35^ We propose a vacancy diffusion model where the swapping of Fe1 can occur via diffusion through different Fe-vacancy sites, as shown in Figure 1c, where the vacancy sites are marked by hollow circles with the corresponding formation energies *E*_f_. Our results verify the fact that the creation of Fe vacancy is most favored at the Fe1 site, as reported by recent experimental studies.^10,13^ Especially, in the case of the UUU configuration, the creation of an Fe1 vacancy is spontaneous with *E*_f_ = −151.0 meV. This again supports that the swapping of Fe1 stabilizes the Fe_5_GeTe_2_ system rather than the scenario when all the Fe1 atoms stay in the same *z* plane (UUU configuration). To have an estimate of the concentration of vacancies in the Fe1 site, we used a (2√3 × 2√3)*R*30° supercell for both UUU and UDU structures to further calculate the evolution of formation energy as a function of the number of Fe1 vacancies. We found that, in both structures, formation of the first Fe1 vacancy in a unit cell is spontaneous, with *E*_f_ = −91.4 and −5.3 meV for UUU and UDU structures, respectively, and therefore, the formation of Fe1 vacancies is possible even at *T* = 0 K. Accordingly, our estimation of the Fe1 vacancy concentration amounts to 1.0 × 10^14^ cm^–2^ (equivalent to 1.7%) in both structures. On the other hand, we found that the formation of more than one vacancy per cell, in Fe1 sites, comes with an energy cost, where positive formation energies of *E*_f_ = 19.1 and 177.5 meV were computed for the formation of the second Fe1 vacancy in UUU and UDU structures, respectively. Using the Arrhenius formula, it was found that, at room temperature, these formation energies correspond to Fe1 vacancy concentrations of 6.5 × 10^14^ cm^–2^ (equivalent to 10.4%) and 1.0 × 10^14^ cm^–2^ (equivalent to 1.7%) for UUU and UDU structures, respectively. It can be noted that the smaller *E*_f_ found in the UUU structure makes it more prone to form vacancies in Fe1 sites at high temperatures, which is another sign that the UUU structure tends to transform to the UDU via rearranging its Fe1 atoms. Moreover, our nudged elastic band (NEB) calculations show that the energy barrier for Fe1 to diffuse to a neighboring vacancy site of Fe2 is only 0.036 eV. Therefore, the diffusion of an Fe atom is possible via different vacancy sites through the unit cell, which can cause the swapping of Fe1 from directly above to below Ge or vice versa.

To confirm that, we reproduced the experimental scenario with a 3^1/2^ × 3^1/2^ periodic pattern of Fe_5_GeTe_2_ and simulated STM images for UDU and UUU configurations; see Figure 1d, upper and lower panels demonstrate UDU and UUU, respectively, where shaded regions show unit cell for these two configurations. In both configurations, Y-shaped features appear along each row connecting three Te atoms to one Fe1 at the center, situated directly above Ge, labeled as Fe1U. STM simulations obtained at *V*_bias_ = −0.05 eV show a dominant contribution from Te p-states appearing as bright ovals (UDU) or circles (UUU). In the case of UDU, the swapping of one of the Fe1 atoms from above to below Ge is reflected in the corresponding STM image showing one dark center with two neighboring bright centers in a given unit cell. On the contrary, the UUU center of each Y appears bright, indicating the presence of all the three Fe1U atoms above Ge for a given unit cell. The shape of Te p-orbitals is asymmetric in UDU and different depending on the presence or absence of Fe1U. This happens because the swapping of one Fe1U causes a trimerization of surface Te atoms and their electronic states, which increases the separation between the surrounding Y-shaped patterns, as observed in the d*I*/d*V* conductance map.^35^ In contrast, the chemical and crystal environments are highly symmetric for UUU (see Figure S1 in the Supporting Information for the structural difference between UDU and UUU). Our simulated STM images are in agreement with the features observed in STM images by Ly et al.^33^ These results re-establish the fact that the formation of a √3 × √3 pattern in Fe_5_GeTe_2_ happens due to the presence of Fe1 split sites.

Similar to the structural properties, magnetism in Fe_5_GeTe_2_ is quite complex and different from other Fe_*n*_GeTe_2_ (*n* = 3, 4) or 2D vdW magnets. To investigate the ground-state magnetic configuration, we considered different magnetic arrangements, for example, ferromagnetic (FM) and ferrimagnetic (FiM) for both UUU and UDU configurations. The lowest energy configuration for UUU is FM, as reported by previous studies.^30,36^ In the case of UDU, the FiM configuration never gets stabilized, and all the moments spontaneously achieve the FM configuration.

Fe atoms present in other Fe_*n*_GeTe_2_ (*n* = 3, 4) systems have a range of spin moments, as already reported in previous studies, where the position of Fe atoms has a mirror reflection along the *c*-axis, and this symmetry is preserved in the magnitude of their magnetic moments as well.^17,37^ We find that the spin moments of five Fe atoms range from 0.11 to 2.59 μ_B_ in the UUU configuration, which are not symmetrically distributed. Also, it is important to note that the moment of an Fe1 species gets highly quenched in UUU, which agrees well with a recent DFT study on Fe_5_GeTe_2_ monolayer.^30^ The distribution of electron occupancy in Fe d-orbitals is responsible for such quenching of magnetic moment for Fe1U; see Table S2 in the Supporting Information.

However, for the UDU configuration, the positions of Fe atoms are partly semisymmetric with respect to Ge along the *c*-axis, and this fact is reflected in the values the of spin moment for Fe5, Fe4, Fe3, Fe2, Fe1U, and Fe1D, which is 2.57, 1.98, 2.08, 2.51, 1.10, and 1.71 μ_B_, respectively, at *T* = 0 K. Our results agree well with neutron powder diffraction data on Fe_5_GeTe_2_ reporting spin moments ranging from 0.8 to 2.6 μ_B_ at 1.5 K.^10^ The magnetic moments in the UDU configuration clearly signify the fact that Fe atoms belong to four different categories, namely, (i) Fe5, Fe2, (ii) Fe4, Fe3, (iii) Fe1D, and (iv) Fe1U. Fe atoms in Fe_5_GeTe_2_ are grouped into three different sites depending on their relative positions as already identified by recent experimental studies,^12,37^ but based on their magnetic properties, Fe atoms are classified into four types. For Fe1 species (both Fe1U and Fe1D), the distribution of electron occupancy between up and down spin channels is different in UDU configuration from UUU; this is the reason why an Fe1 species regains its moment. Densities of states projected on Fe d-orbitals for each Fe species are shown in Figure S2 in the Supporting Information. Different moments on Fe1U and Fe1D are due to their different periodicity in √3 × √3 cell and also different coordination with their neighbors. For example, the cell parameter for Fe1D sublattice is 6.99 Å, which is larger than Fe1U (4.00 Å). Also, Fe1D and Fe1U species have one and three first nearest neighbors at a distance of 2.46 and 2.44 Å, respectively. Therefore, Fe1U has a larger effective coordination number than Fe1D (see Table S3). It is known that the magnetic moment of Fe always increases with cell dimension and decreases with increasing coordination number.^38,39^ Note, in the case of DUD, which is the energetically degenerate partner of UDU, moments of Fe1U and Fe1D just get interchanged and become 1.72 and 1.12 μ_B_, respectively, since their crystal environment becomes the exact opposite of a UDU configuration.

To investigate the magnetic interactions among different types of Fe atoms in a √3 × √3 UDU configuration, we calculated the isotropic symmetric exchange coupling parameters *J*_*ij*_ between the *i*th and *j*th atoms. Figure 2a,b shows exchange interactions between the *i*th and *j*th Fe atoms, considering the *i*th atom as Fe1U and Fe5, respectively. The first neighbor exchange interaction of Fe1U with both Fe2 and Fe3 is strongly ferromagnetic (FM), while Fe1U–Fe4 and Fe1U–Fe5 interactions are weakly FM. When the *i*th site is Fe5, the dominating (first neighbor) exchange interactions with Fe4 and Fe3 species are strongly FM. It is important to note that all in-plane *J*_*ij*_ couplings between the same species are AFM, while for Fe1U, this AFM interaction is quite significant. The overall nature of *J*_*ij*_ couplings for the other Fe species, for example, *i* = Fe4, Fe3, Fe2, and Fe1D, are quite similar. The first neighbor interactions are strongly FM for *i* ≠ *j*, while the interaction between the same species is AFM. The *J*_*ij*_ values for each Fe species in both UDU and UUU configurations are plotted in Figure S4 in the Supporting Information, which shows an enhancement of FM exchange couplings in the UDU configuration.

**Figure 2:**
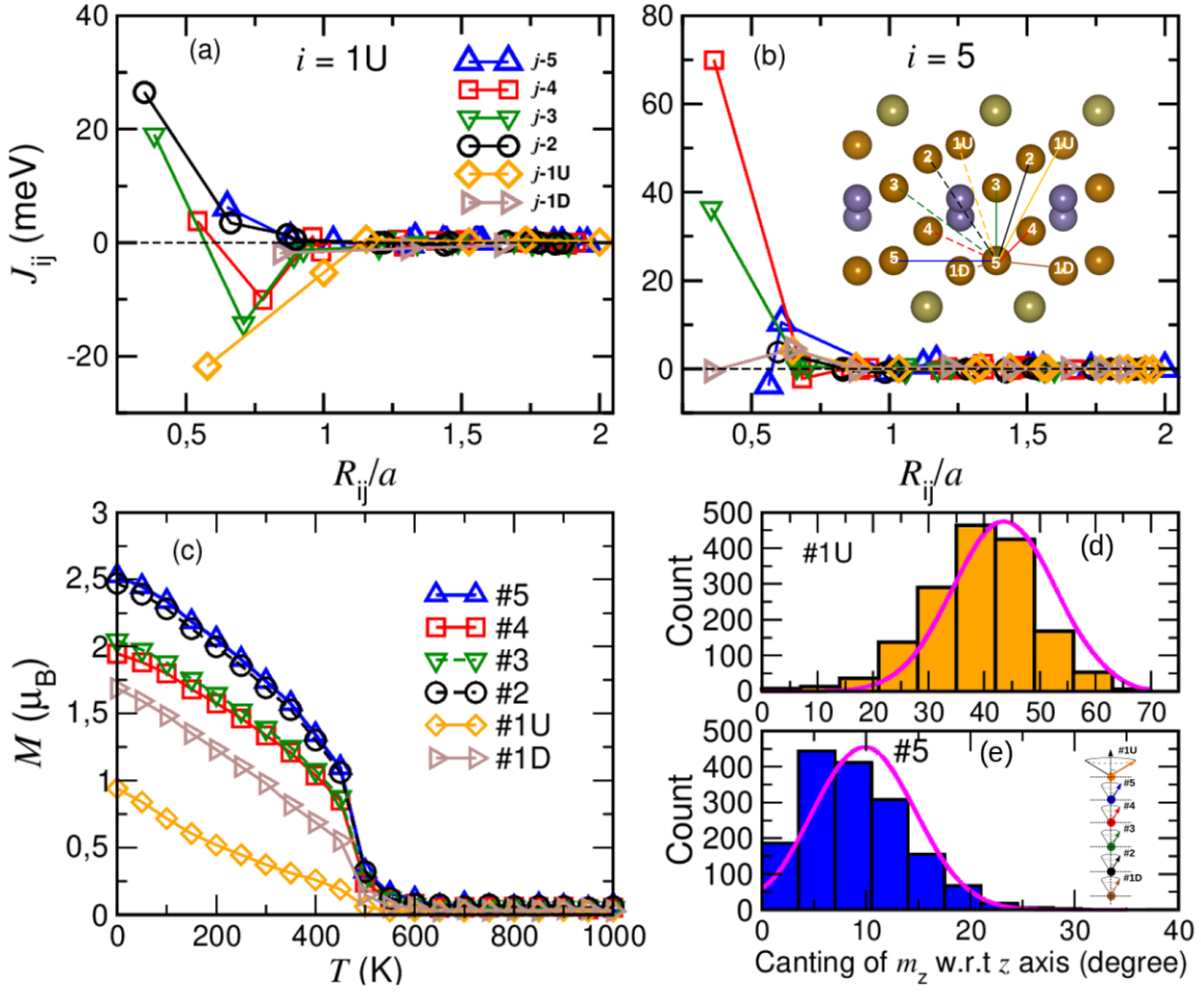
Magnetic exchange parameters *J*_*ij*_ with neighboring distance *R*_ij_/*a* for UDU, *i*th site is (a) Fe1–U and (b) Fe5 (inset shows exchange interaction paths), *J*_*ij*_ > 0: FM coupling. In each case, the *J*_*ij*_ values are multiplied by the corresponding coordination numbers. (c) Magnetization (*M*) for each Fe sublattice as a function of temperature (*T*). Histograms showing canting of the *z*-component of magnetic moment *m*_*z*_ wrt easy axis for Fe5 and Fe1U at 0 K in (d, e), respectively. (e, inset) shows a schematic representation of canting angles of *m*_*z*_ wrt *z* for each Fe species.

Although from collinear total energy calculations, the UDU configuration is found to be spontaneously FM, some *J*_*ij*_ couplings are AFM in nature. From our calculations, it is found that the Fe_5_GeTe_2_ monolayer has weak MAE along the out-of-plane direction in a UDU configuration, with a magnitude of 0.021 meV/Fe. A weak out-of-plane anisotropy in bulk Fe_5_GeTe_2_ has also been reported in recent experiments.^34^ For the UUU configuration, the in-plane anisotropy is calculated to be 0.112 meV/Fe, agreeing well with the previously reported result.^36^ It is important to note that the MAE for Fe_5_GeTe_2_ is quite small compared to other 2D vdW magnets, for example, Fe_3_GeTe_2_,^17,36^ Fe_4_GeTe_2_,^17,36^ and CrI_3_.^21,40^ Compared to UDU, *J*_*ij*_ interactions for a given *i*–*j* pair is weaker in UUU, but the overall trend remains almost similar, except for Fe1U. A significant difference in *J*_*ij*_ is observed for Fe1U due to its quenched moment. Figure S4 in the Supporting Information shows the comparison in *J*_*ij*_ couplings between UDU and UUU configurations.

In order to obtain the Curie temperature *T*_C_, we modeled the Heisenberg Hamiltonian with isotropic exchange interactions and single-site anisotropy, details of which are given in the Methods section in the Supporting Information. *T*_C_ for Fe_5_GeTe_2_ monolayer in the UDU configuration obtained from Monte Carlo simulation is 492 K, shown in Figure 2c. However, *T*_C_ calculated in this study is overestimated compared to the experimental value.^10,12,13,15,33^ This difference might arise due to the mixture of UDU and UUU configurations in experimental samples or some other complex arrangements of different domains of these two configurations in the presence of a vacancy or some impurities.^10,33^ Apart from the consideration of these complex structures, proper treatment of electron correlation effects within dynamical mean-field theory can also play an important role. We are pursuing this study and will report on it in future communication.

Similar to the spin moment values, the magnetization (*M*) versus temperature (*T*) behavior for the UDU configuration also supports the classification of five Fe atoms into four different categories. The *M* versus *T* behavior is strikingly different for an Fe1U sublattice than for others. This particular Fe species primarily governs the magnetic properties and is responsible for anomalous magnetic behavior in a low-temperature regime.^10,13,15,33^ Though the easy axis of magnetization for a UDU monolayer is along the out-of-plane direction, the *z*-component of magnetization vector (*m*_*z*_) for Fe1U is significantly deviated (with mean value of ∼42°) wrt easy axis of magnetization, that is, *z*-direction even at *T* = 0 K and its close vicinity. Such behavior of Fe1U can be related to canted moments or spin fluctuations observed in recent experiments for bulk Fe_5_GeTe_2_.^15,33^ The Fe1 species is special from others because not only *T*_C_ but also physical properties like Hall and Seebeck coefficients are impacted by the magnetic ordering of Fe1 according to experimental data.^12^

On the contrary, in the case of Fe5, the fluctuation of *m*_*z*_ wrt the *z*-axis is rather small (∼12°). Figure 2d,e shows the number of Fe1U and Fe5 atoms present in a large supercell with average canting angles of 42° and 12°, respectively, from the easy axis of magnetization. The inset in Figure 2e shows the canting of *m*_*z*_ wrt *z* for each Fe sublattice. In addition, to see the orientation of spin moments at finite temperatures, the angular distribution between *m*_*z*_ and *z* at *T* = 100 and 50 K for Fe1U and Fe5 is plotted in Figure S6 in the Supporting Information. Deviation of *m*_*z*_ wrt the easy axis is larger for Fe1U than for Fe5 at a finite temperature supporting the large fluctuation of Fe1 moments reported by May et al.^10,12^ The deviation of *m*_*z*_ for other Fe species (Fe2, Fe3, Fe4, and Fe1D) wrt *z* remains the same as that for Fe5, that is, 12°; see Figure S5 in the Supporting Information.

In the case of the UUU configuration, which has a lower symmetry compared to the UDU configuration, each Fe species behaves differently, as seen from the *M* versus *T* result shown in Figure S8 in the Supporting Information. Also, because of a stronger in-plane MAE, the deviation of the in-plane component *m*_*x*_ (or *m*_*y*_) of the magnetic moment vector for each Fe species is ∼7° from the easy axis of magnetization, which is smaller than the same for the UDU configuration. As already discussed, the moment of Fe1 gets hugely quenched to 0.11 μ_B_ for the UUU configuration. This quenching of magnetic moment of Fe1 affects the *T*_C_ of the monolayer. As a result, we find *T*_C_ to be 390 K for the UUU configuration. The spin moments of Fe2, Fe3, and Fe4 sublattices in the UUU configuration differ from the same in the UDU configuration, while the moment of Fe5 remains almost unchanged. Spin and orbital moments for each Fe species in both UDU and UUU configurations are reported in Table S1 in the Supporting Information. It is important to note that Fe1D has the largest orbital moment. The importance of the Dzyaloshinskii-Moriya (DM) interaction for this system has been mentioned in literature. It would be interesting to study the strength of this interaction in various scenarios.

We also find that the presence of an Fe vacancy (6.67%) influences *J*_*ij*_ interactions (see Figure S9) and hence the value of *T*_C_. For example, because of the absence of one Fe1U out of three in the √3 × √3 unit cell, the remaining Fe1 atoms regain their moment to 1.68 μ_B_. The presence of an Fe1 vacancy increases the moments of Fe2, Fe3, and Fe4 sublattices wrt pristine UUU, whereas the Fe5 moment remains unchanged proving its robustness. As a result, *T*_C_ increases to 435 K. A similar trend has been observed in experiments,^10,15^ where *T*_C_ is observed to be higher for a higher Fe vacancy concentration. However, *T*_C_ with an Fe1 vacancy concentration of 6.67% falls between UDU and UUU configurations; see Figure S7 in the Supporting Information. We also find that the *M* versus *T* behavior follows the same trend as that for the spin moments; for example, Fe5 and Fe2 sublattices are equivalent, while other Fe sublattices behave differently. The presence of an Fe1 vacancy yields a very weak MAE along the out-of-plane direction. There is an ∼12° deviation of the *m*_*z*_ component from the *z*-axis for Fe1U and Fe5 at 0 K (Figure S10 in the Supporting Information).

We studied the electronic properties of UDU and UUU configurations to understand the consequence of swapping as shown in Figure 3. Figure 3a shows DOS projected on Fe5 and Fe1U in the UUU configuration where the spin moments of Fe5 and Fe1U are 0.11 and 2.57 μ_B_, respectively. The bottom panel shows DOS for Fe1 situated above (Fe1U) and below (Fe1D) Ge. The main difference between the atom-projected DOS of Fe1U (black curve) and Fe1D (red curve) in UDU is the presence of a large peak in the spin-down channel of Fe1D close to the Fermi level (*E*_F_), which comes from the d_*z*_^2^ orbital (see Figure S2) and is also evident from the magnetization density plot at *E* – *E*_F_ = −0.24 eV. Spin-polarized DOS for Ge in UUU and UDU configurations are plotted in (c) and (d). In (d), Ge–U and Ge–D correspond to two split sites of Ge. A sharp feature appearing in the PDOS of Ge–U comes from the p_*z*_ orbital (see Figure S3). A comparison between the total DOSs of UUU and UDU configurations shows that the UUU configuration has more fragmented features.

**Figure 3:**
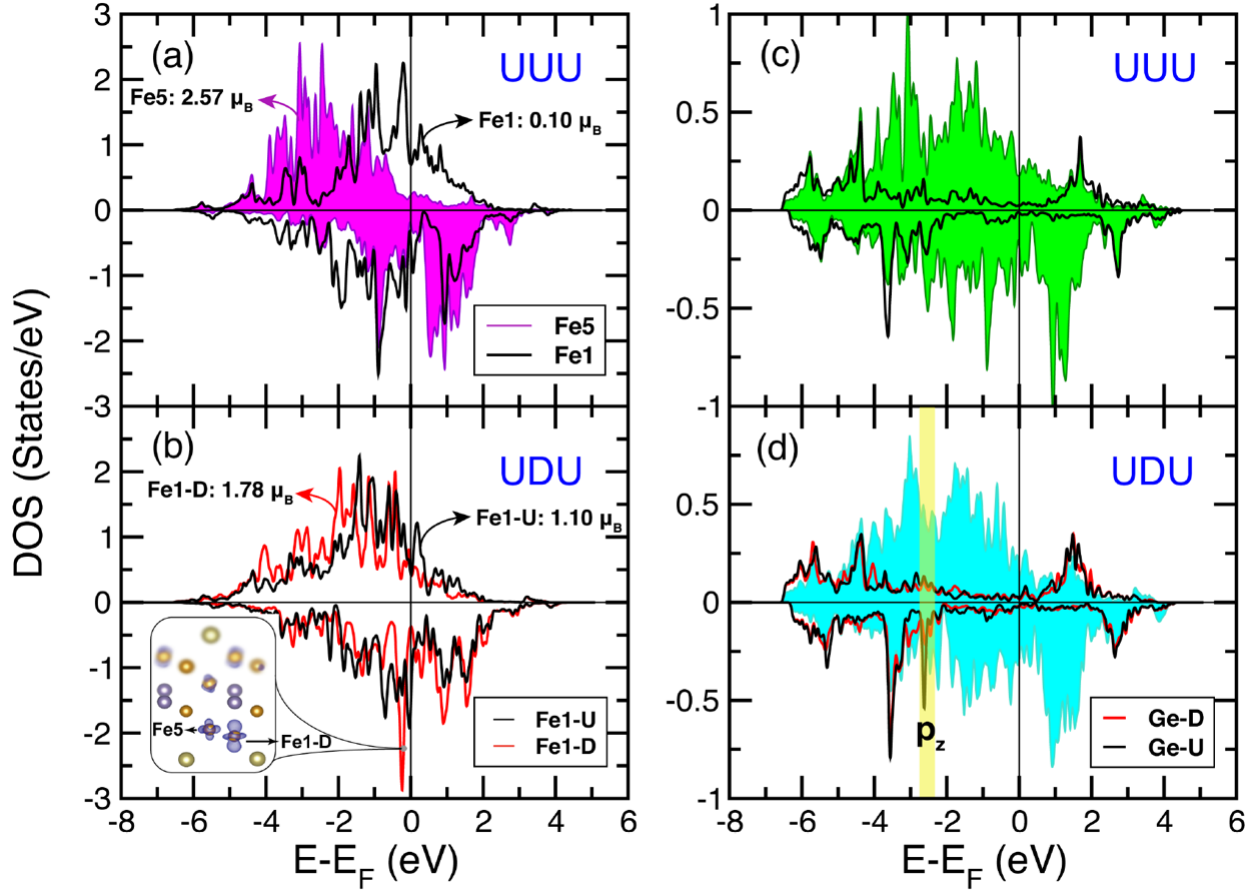
Spin-polarized PDOS for (a) Fe5 (filled magenta) and Fe1 (black) in UUU. (b) PDOS for Fe1U (black) and Fe1D (red) in UDU. The sharp peak in the PDOS of Fe1D close to *E*_F_ comes from *d*_*z*_^2^, as shown in the magnetization density obtained at *E* – *E*_F_ = −0.24 eV. (inset) Blue lobes correspond to a negative sign for magnetization density obtained at isosurface value = 0.004 e/bohr^3^. The values correspond to spin moments for the corresponding Fe sublattices in UUU (a) and UDU (b) configurations. PDOS for Ge in (c) UUU and (d) UDU, solid background shows total DOS of (c) UUU and (d) UDU. (d) PDOS for Ge split sites, e.g., Ge-up (black) and Ge-down (red) are plotted. The sharp peak present in the spin-down channel of Ge–U comes from *p*_*z*_, highlighted in a light yellow stripe.

In conclusion, using DFT calculations, we show that the swapping of an Fe1 atom occurs via an Fe vacancy-mediated diffusion causing Fe1 split sites, which gives rise to a √3 × √3 pattern, as observed in experiments. We find that the magnetic behavior of Fe1 is very different from other Fe atoms, which primarily governs the magnetic properties of Fe_5_GeTe_2_. Even though the *T*-dependent *M* behavior observed in experiments is not properly visible in our present work, this study is important to understand the structural and magnetic properties of Fe_5_GeTe_2_, a promising candidate for topology-based spintronic devices. In our future study, we will use dynamical mean-field theory for treating electron correlation in these metallic systems.

## Supporting Information Available

Methods and computational details, atomic structures showing top views for UDU and UUU, projected density of states plots for different Fe species in UDU, comparison in exchange interaction between UDU and UUU configurations for each Fe species, canting of spin moments wrt easy axis of magnetization for different Fe species in UDU at *T* = 0 K, deviation of *m*_*z*_ for Fe1U and Fe5 wrt easy (*z*) axis of magnetization at finite temperature, comparison in *M* versus *T* behavior among UDU, UUU without and with Fe1 vacancy site, *M* versus *T* behavior for UUU projecting on different Fe sublattices, magnetic properties of UUU with Fe1 vacancy, spin and orbital moments of each Fe species in UDU and UUU configurations, electron density distribution in up spin and down spin channels for each Fe species in UDU and UUU, nearest neighbor distance and coordination number for Fe atoms in UDU and UUU (PDF)
