# Supplementary material for: Unusual Magnetic Features in Two-Dimensional Fe5GeTe2 Induced by Structural Reconstructions
Source: J Phys Chem Lett. 2022 May 26;13(22):4877–83. doi: 10.1021/acs.jpclett.2c00692 (PMC9189922; doi:10.1021/acs.jpclett.2c00692)
Supplement: Supplementary file 1 — jz2c00692_si_001.pdf [file jz2c00692_si_001.pdf]

Supplementary Material: Unusual Magnetic Features in Two-Dimensional Fe_5GeTe_2 Induced by Structural Reconstructions

Soheil Ershadrad[§], Sukanya Ghosh[§], Duo Wang, Yaroslav Kvashnin, and Biplab Sanyal[*]

Department of Physics and Astronomy, Uppsala University, Box-516, 75120 Uppsala, Sweden

E-mail: Biplab.Sanyal@physics.uu.se

Methods and Computational Details

DFT calculations of structural, electronic, and magnetic properties were done via Vienna Ab initio Simulation Package (VASP).[1,2] The exchange-correlation potential was approximated by the generalized gradient approximation (GGA) with Perdew, Burke, and Ernzerhof (PBE) functional.[3] For integration in the Brillouin zone, we find a 20×20×1 k-point grid in the Monkhorst-Pack scheme[4] for a 1×1 cell of Fe_5GeTe_2 monolayer is sufficient enough to produce converged structural, electronic, and magnetic properties. The equilibrium lattice constants and atomic positions were optimized through energy minimization, using the conjugate gradient method up to the point that force components on each atom were below 0.01 eV/Å. The interaction between periodic images along the z-axis was minimized by adding a vacuum spacing of at least 15 Å. We considered the convergence of total potential to vacuum level to determine the most efficient vacuum space thickness. Saddle points and minimum energy diffusion paths between two stable states were found through climbing image Nudged Elastic Band (NEB) calculations using VTST tools.[5] Number of intermediate images along the diffusion path was considered to be 5 and the spring force between them was set to be -5 eV/Å^2. The structures optimized with VASP were used for the calculations of inter-atomic magnetic exchange parameters (Jij). For these calculations, magnetic force theorem (MFT),[6] and full-potential linear muffin-tin orbital (FP-LMTO) method, implemented in RSPt code,[7] were utilized. A dense k-points grid of 18×18×1 was used in all RSPt calculations for $\sqrt{3} \times \sqrt{3}$ unit cell. The extracted J_{ij}s were implemented in a Heisenberg Hamiltonian to calculate the magnetic ordering temperatures by performing classical Monte Carlo (MC) simulations via UppASD code.[8] To achieve properly averaged properties, calculations were done for five ensembles in supercell with size 40×40×1 and periodic boundary condition. Simulation of scanning tunneling microscopy (STM) images is performed using the Tersoff-Hamann approach in constant-height mode.[9]

Vacancy formation energies are calculated based on the following expression,

$$E_f = E_{Fe_{5-x}GeTe_2} - E_{Fe_5GeTe_2} + E_{Bcc-Fe} \tag{1}$$

where, E_f is the vacancy formation energy, and the terms on the right-hand side are the total energy of the vacant crystal, the total energy of the stoichiometric crystal, and energy of one Fe atom in bulk BCC form, respectively.

In order to obtain the Curie temperature T_{C}, Monte Carlo simulations are performed using the following spin-Hamiltonian:

$$H = -\sum_{i \neq j} J_{ij} e_i \cdot e_j - \sum_i K_i (e_i^z)^2, \tag{2}$$

where J_{ij} is the symmetric isotropic Heisenberg exchange coupling between site i and j, e_i denotes the unit vector along the magnetic moment at the ith site, $J_{ij} > 0$ implies ferromagnetic coupling. K_i is the strength of single ion uniaxial magnetocrystalline anisotropy energy (MAE) for site i, which is assumed to be the same for each Fe atom and obtained by dividing the total MAE/cell by the number of Fe atoms present in the unit cell.

Methodology for J_{ij} calculations

The isotropic symmetric exchange interactions Jij are calculated within the full-potential linearized muffin tin orbital (FPLMTO) basis implemented in the Rspt code.

From the LMTO basis, one can construct the Bloch sums to solve the DFT eigenvalue problem and subsequently for the one-electron Green's function. We have considered Löwdin orthonormalized LMTO-"ORT", which are not very localized due to their long decaying tail, and are more physical for metallic systems like Fe_5GeTe_2. For a detailed description of the shape of various local orbitals used in Rspt, please see Ref. 10. ORT used in our calculations has been constructed from the original LMTO basis functions performing a k-point-wise orthonormalization.[10] In this way, the k-space dependence in ORT has been removed.

J_{ij} can be extracted from the Green function obtained from the LMTO basis for the atomic site i. The generalized expression for the intersite exchange parameters is given by:

$$J_{ij} = \frac{T}{4} \sum_n [\hat{\Delta}_i(i\omega_n) \hat{G}^{\uparrow}_{ij} \hat{\Delta}_j(i\omega_n) \hat{G}^{\downarrow}_{ji}], \tag{3}$$

where the trace is performed over the orbital degrees of freedom. T and $\omega_n = 2(2n+1)$ are the temperature and the nth fermionic Matsubara frequency. $\hat{G}^{\sigma}_{ij}$ is the intersite Green's function between sites i and j and projected over a given spin σ. The term Δ_i gives the exchange splitting at site i, obtained using spin and site-projected Kohn-Sham Hamiltonian. Therefore all the terms appearing in the expression of J_{ij} parameters are obtained in orbital and spin-space and there is no k-point dependence.

We have checked the convergence of spin moments and exchange interactions with the k-point grid and Matsubara frequency ω_n, since the precision of the computed J_{ij} parameters is controlled by the number of k-points and Matsubara frequencies.[7] The spin moments are converged up to $\approx 10^{-3}\mu_B$ w.r.t k-point grid. The trend and sign of J_{ij} parameters remain essentially unchanged with an increase in the k-point grid with minimal change in their magnitudes. Our results show the J_{ij} values deviate at most by $\approx 8\%$ with change in the k-grid from $20 \times 20 \times 1$ to $40 \times 40 \times 1$ for both UUU and UDU configurations. We have also checked the convergence of J_{ij} w.r.t Matsubara frequencies by increasing the number of frequencies n (see eq. 3) from 1500 to 3000, which in turn doubles the Matsubara frequency ω_n, and we found the deviation of J_{ij} values is $\approx 5\%$.

Top views for UDU and UUU

Fig. S1 shows top views of UDU (a) and UUU (b) configurations. Side of triangles formed by surface Te atoms are smaller for UDU (3.98 Å) than UUU (4.01 Å), indicating Te atoms are more closely spaced in the case of UDU while forming trimers, appeared as “Y" shaped features in simulated STM images shown in Fig. 1d. Also, the distance between Fe1U situated

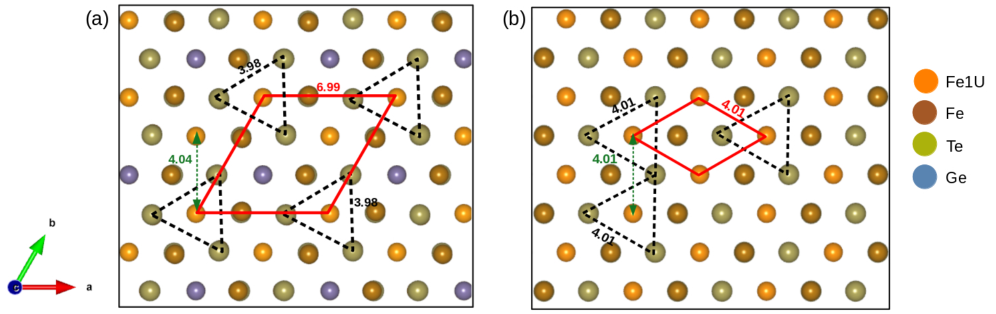

Figure S1: Top views showing (a) UDU and (b) UUU configurations. Red rhombus shows unit cell for both configurations. The triangles with dashed black lines show trimers formed by Te atoms. The green arrow shows the shortest distance between Fe1U atoms situated at the centre of each triangle for each structure. Distances showed in (a) and (b) are in units of Å. Orange, brown, dark green, and purple spheres show Fe1U, Fe, Te, and Ge atoms, respectively.

at the centre of the triangles is slightly larger for UDU (4.04 Å) than UUU (4.01 Å). The red rhombus shows the unit cell for each configuration. The top view of UDU explains the formation of $\sqrt{3} \times \sqrt{3}$ pattern observed in STM images and the dI/dV conductance map reported by recent experimental studies.[11–13]

Projected density of states plots for different Fe species

Fig. S2 shows DOS plots for each Fe species projecting on Fe$-d$ orbitals in UDU configuration. Dominating contribution arises from d_{z^2} orbital for each Fe species.

Fig. S3 shows DOS plots for different Ge split sites in UDU structure, projected on Ge$-p$ orbitals, where p_z of Ge has the maximum contribution.

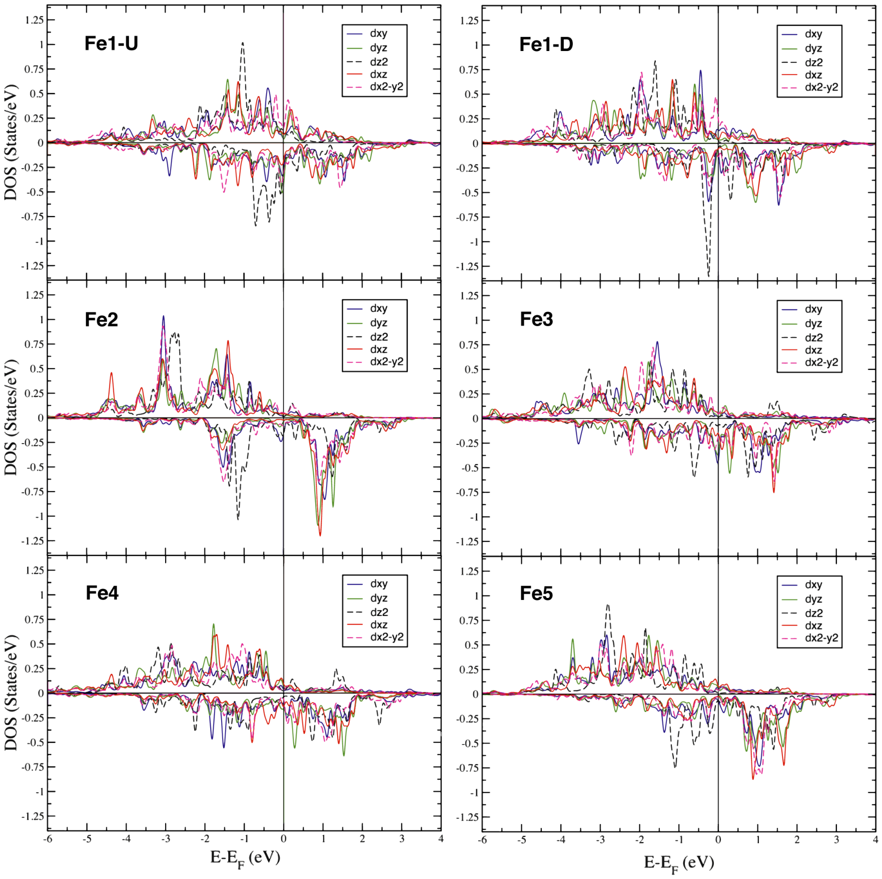

Figure S2: Fe-d orbital projected density of states (PDOS) of different Fe species in UDU structure, where the solid vertical line shows Fermi level. Blue, green, black, red, and magenta curves show $d_{xy}, d_{yz}, d_{z^2}, d_{xz}$, and $d_{x^2-y^2}$ orbitals, respectively.

Comparison in exchange interactions between UDU and UUU

Fig. S4 shows isotropic symmetric exchange interactions between different ith and jth sites for UDU (a)–(f) and UUU (g)–(k) configurations. It is important to note that for a given ith and jth pair J_{ij}, coupling is always more FM for $i \neq j$, especially in the case of UDU. More interestingly, the nearest neighbor J_{ij} interaction for 1U-1U is strongly AFM for UDU, while such interaction is relatively weak for UUU because of the quenched moment on Fe1U. For each ith component, the nature of dominating J_{ij} couplings are quite similar in nature for UDU and UUU. However, the strength of the interaction is suppressed by $\sim 20-10$ meV

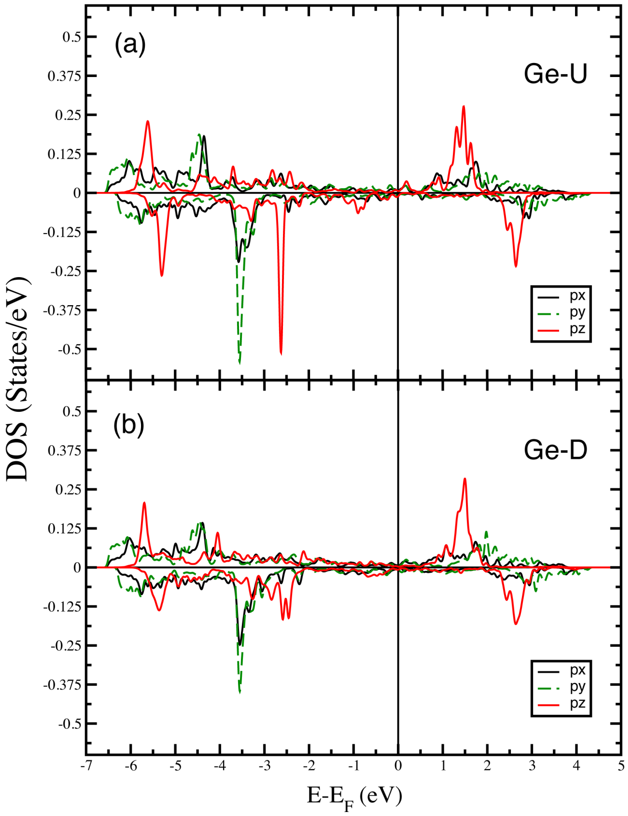

Figure S3: Ge-p orbital projected density of states (PDOS) of different split sites in UDU structure, (a) Ge-U and (b) Ge-D, where solid vertical line shows Fermi level. Blue, green, black, red and magenta curves show $d_{xy}, d_{yz}, d_{z^2}, d_{xz}$ and $d_{x^2-y^2}$ orbitals, respectively.

in UUU.

Canting of spin moments w.r.t. easy axis

Fig. S5 shows canting of m_z component w.r.t. z, i.e., the easy axis of magnetization, for (a) Fe4, (b) Fe3, (c) Fe2, and (d) Fe1D in UDU configuration at 0 K temperature. Deviation of m_z is found to be $\approx 12^0$ for these Fe sublattices.

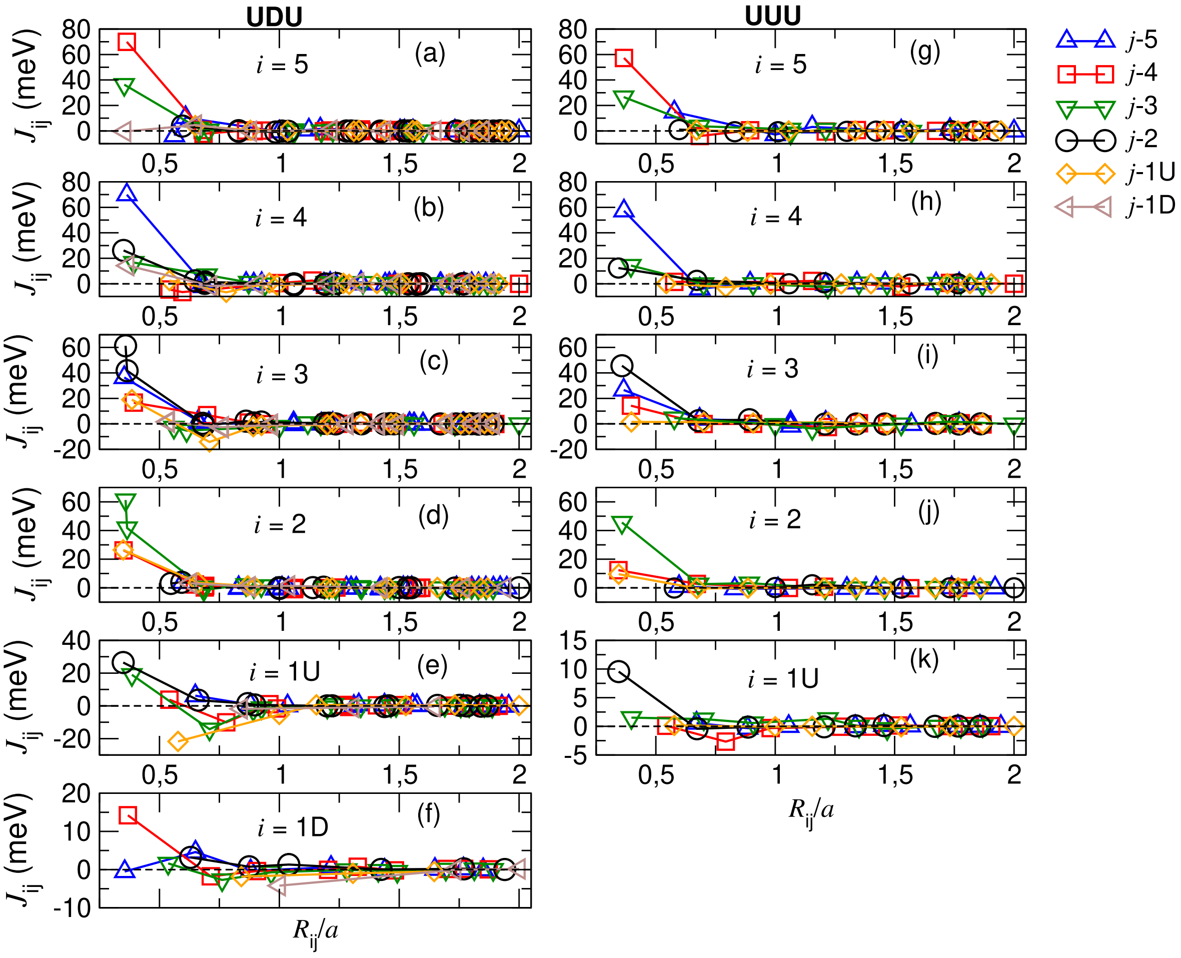

Figure S4: Magnetic exchange parameters J_{ij} corresponding to i-th Fe atom as a function of its distance with neighboring j-th Fe atoms, R_{ij}/a, for Fe_5GeTe_2 monolayer in (a)–(f) UDU and (g)–(k) UUU configurations. In each case, the J_{ij} values are multiplied by the corresponding coordination numbers.

Fluctuation of spins w.r.t. easy axis at finite temperature for Fe1U and Fe5

Fig. 6 shows the fluctuation of magnetic moment, more precisely, m_z component of the moment for Fe1U and Fe5 w.r.t. easy axis of magnetization z at 50 K (a), (b), and 100 K (c), (d). It is important to note that the distribution of spins deviated from z-axis gets broader with an increase in temperature for both Fe1U and Fe5. However, this deviation is quite prominent for Fe1U, supporting experimental data reporting large fluctuation of Fe1 spin moments up to the temperature $\sim$ 100 K.[12]

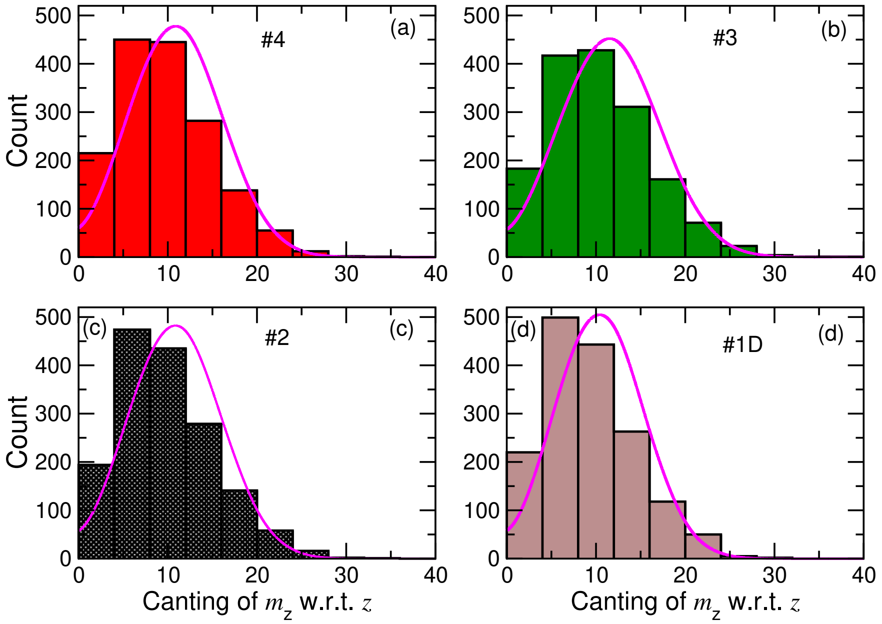

Figure S5: Canting of magnetization vector m_z w.r.t z direction for (a) Fe4, (b) Fe3, (c) Fe2 and (d) Fe1D at 0 K in UDU configuration.

M vs. T behavior for UDU and UUU without and with Fe1 vacancy

Fig. S7 shows M/M_S vs. temperature (T) behavior for UDU, UUU without and with Fe1 vacancy (concentration 6.67%). T_C = 390 K, 435 K and 492 K for UDU, UUU without and with Fe1 vacancy, respectively. The T_C for each system is obtained from the cumulant crossing method that originally was suggested by Binder, which is a very powerful and useful technique.[8]

M vs. T behavior projecting on Fe sublattices in UUU

M vs. T plot in Fig. S8 shows Fe sublattices fall into five different categories as all the five Fe species behave differently, with $T_C = 390$ K for UUU configuration. Inset shows canting of m_x for Fe5 w.r.t. x-axis, i.e., the easy axis of magnetization.

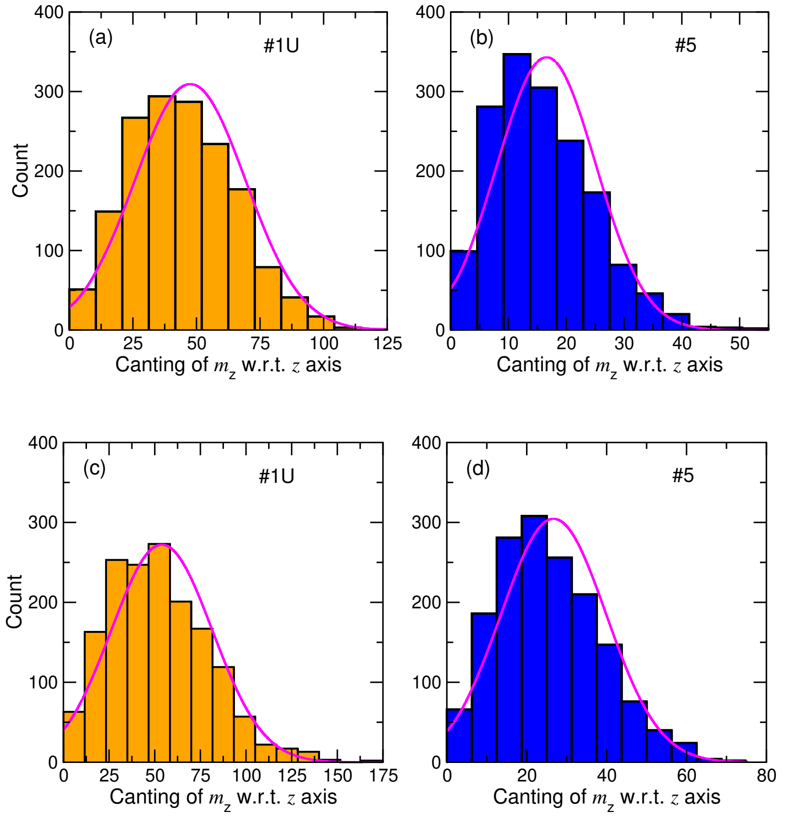

Figure 6: Canting of magnetization vector m_z w.r.t z direction for Fe1U and Fe5 at (a), (b) 50 K and (c), (d) 100 K in UDU configuration.

Magnetic properties of UUU with Fe1 vacancy

Fig. S9 shows J_{ij} couplings when one of the Fe1 is removed from $\sqrt{3} \times \sqrt{3}$ cell of UUU, thus creating Fe-vacancy of 6.67% concentration. Exchange couplings for each ith Fe species are more FM in the presence of Fe1 vacancy than pristine UUU. It is important to note that for i =Fe1U J_{ij} interactions are more robust than pristine UUU, because remaining Fe1U atoms regain their moment with Fe1 vacancy. Also, exchange interaction is AFM when $i = j$ =Fe1U, a similar feature exists for UDU but with stronger strength (see Fig. 2).

M vs T for this system is plotted in Fig. S10(a). Deviation of m_z for Fe1U and Fe5 w.r.t. easy (z) axis are plotted in Fig. S10(b) and (c), respectively.

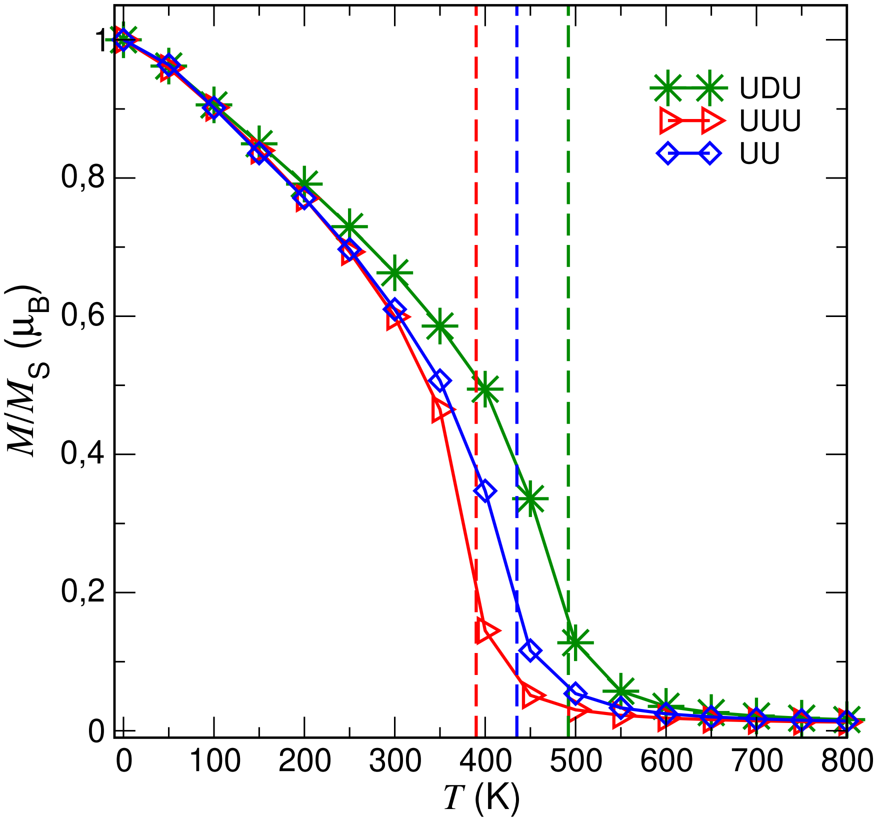

Figure S7: (a) M/M_S vs. temperature (T) plot for UDU and UUU configurations summed over all Fe species present in 40×40 supercell. The dashed vertical lines show T_C for corresponding systems obtained from the cumulant crossing method.

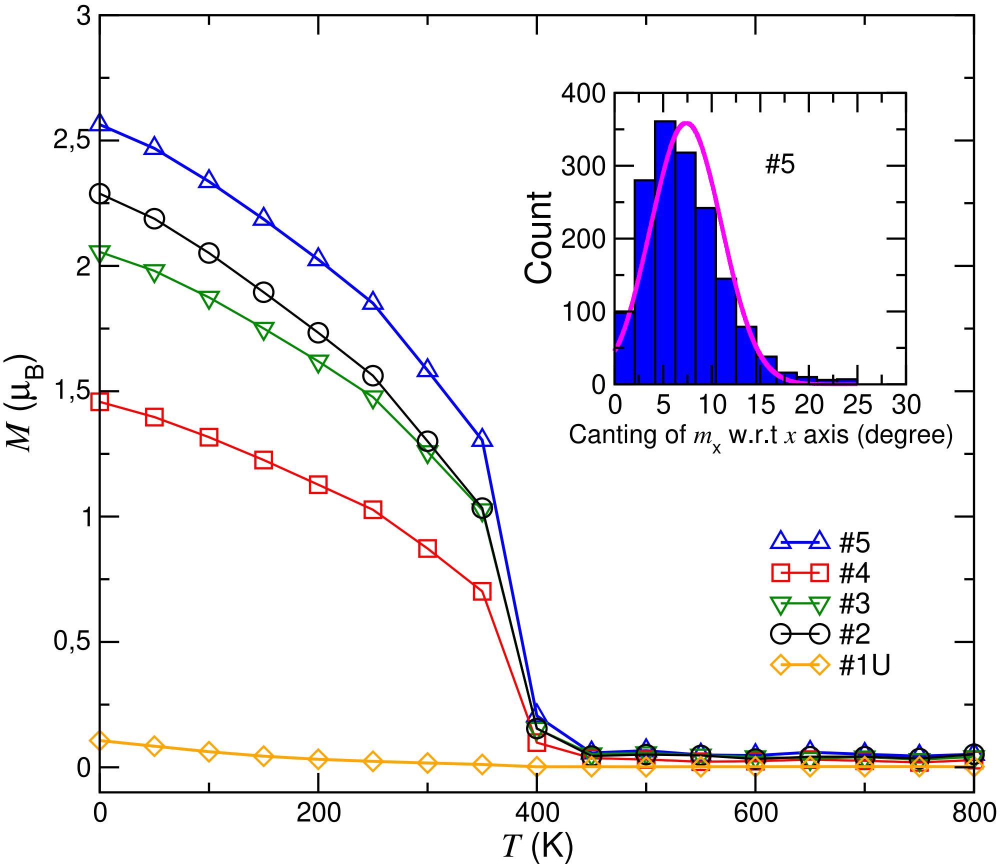

Figure S8: (a) Magnetic moment (M) for each Fe sub-lattice as a function of temperature (T) in UUU configuration. Inset shows canting of magnetization vector m_x w.r.t x-axis for Fe5 at 0 K.

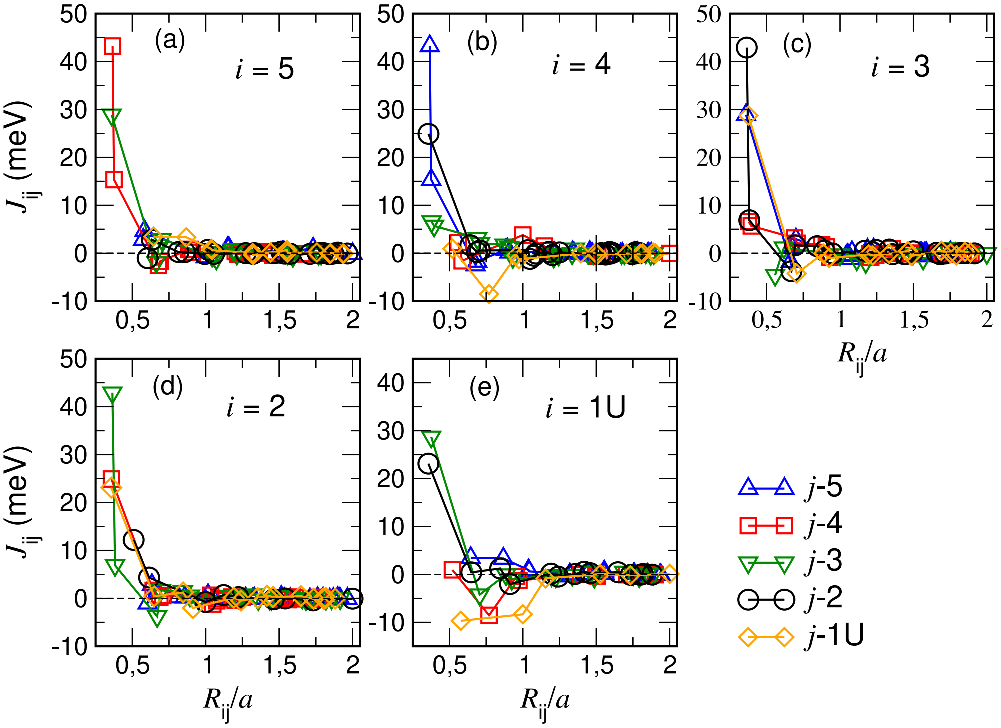

Figure S9: Magnetic exchange parameters J_{ij} corresponding to i-th Fe atom as a function of its distance with neighboring j-th Fe atoms, R_{ij}/a, for Fe_5GeTe_2 monolayer with Fe1U vacancy in UU configuration. In each case, the J_{ij} values are multiplied by the corresponding coordination numbers.

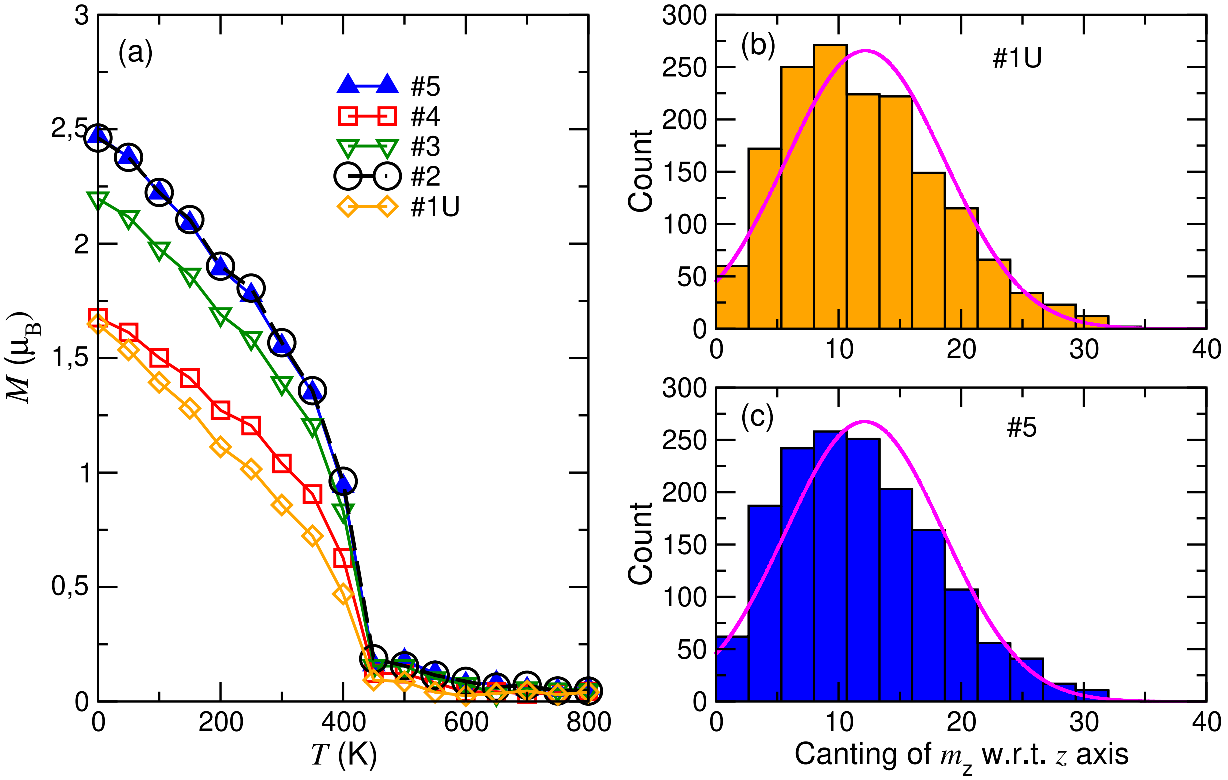

Figure S10: (a) Magnetic moment (M) for each Fe sub-lattice as a function of temperature (T) when one of the Fe1 is removed from the unit cell of UUU. (b) and (c) show canting of m_z w.r.t z-axis for Fe1U and Fe5, respectively, at 0 K.

Table S1: Spin, orbital, and total moments for each Fe species in UDU and UUU configurations obtained from fully relativistic calculations with the inclusion of spin-orbit coupling.

System	Spin axis	Fe species	Spin moment (μ_B)	Orbital moment (μ_B)	Total moment (μ_B)
UDU	001 (easy axis)	Fe5	2.570	0.070	2.641
		Fe4	1.977	0.046	2.024
		Fe3	2.074	0.042	2.115
		Fe2	2.505	0.057	2.562
		Fe1U	1.101	0.041	1.142
		Fe1D	1.787	0.104	1.892
	100	Fe5	2.568	0.067	2.635
		Fe4	1.981	0.057	2.037
		Fe3	2.075	0.057	2.135
		Fe2	2.505	0.062	2.568
		Fe1U	1.108	0.046	1.156
		Fe1D	1.776	0.071	1.848
UUU	001	Fe5	2.575	0.071	2.645
		Fe4	1.476	0.032	1.508
		Fe3	2.055	0.029	2.084
		Fe2	2.295	0.044	2.340
		Fe1U	0.094	-0.022	0.072
	100 (easy axis)	Fe5	2.575	0.078	2.652
		Fe4	1.474	0.038	1.513
		Fe3	2.055	0.058	2.113
		Fe2	2.293	0.049	2.342
		Fe1U	0.109	-0.006	0.103

Table S2: Difference in occupancy for Fe-d orbitals between up (↑) and down (↓) spin channels, where $N = \int_{-\infty}^{E_F} n(E)dE$. Orbital projected magnetic moments are reported for both UDU and UUU configurations.

System	Fe atom	N(dz2↑ − ↓)	N(dzx↑ − ↓)	N(dzy↑ − ↓)	N(dx2-y2↑ − ↓)	N(dxy↑ − ↓)
UDU	1U	0.19	0.25	0.25	0.21	0.21
	1D	0.37	0.38	0.38	0.34	0.34
	2	0.36	0.64	0.63	0.43	0.48
	3	0.40	0.47	0.46	0.39	0.41
	4	0.37	0.45	0.45	0.41	0.36
	5	0.38	0.60	0.60	0.55	0.48
UUU	1	-0.021	0.123	0.123	-0.032	-0.032
	2	0.29	0.63	0.64	0.40	0.41
	3	0.45	0.46	0.46	0.39	0.39
	4	0.26	0.36	0.35	0.31	0.32
	5	0.45	0.48	0.49	0.59	0.60

Table S3: Distance (in Å unit) between neighbors for different Fe species is reported, where the number of neighbors is given in parenthesis.

Species	Configurations			
	UDU		UUU	
	1st NN	2nd NN	1st NN	2nd NN
Fe1U	2.44 (3)	2.46 (1)	2.39 (3)	2.45 (1)
Fe1D	2.46 (1)	2.48 (3)	–	–
Fe2	2.44 (2)	2.45 (1)	2.38 (1)	2.39 (3)
Fe3	2.47 (3)	2.50 (1)	2.48 (3)	2.50 (3)
Fe4	2.45 (4)	2.54 (2)	2.37 (3)	2.38 (1)
Fe5	2.47 (1)	2.48 (1)	2.54 (4)	2.73 (3)

References

(1) Kresse, G.; Joubert, D. From Ultrasoft Pseudopotentials to the Projector Augmented-Wave Method. *Phys. Rev. B* **1999**, *59*, 1758.

(2) Kresse, G.; Hafner, J. Norm-Conserving and Ultrasoft Pseudopotentials for First-Row and Transition Elements. *J. Phys. Condens. Matter* **1994**, *6*, 8245.

(3) Perdew, J. P.; Burke, K.; Ernzerhof, M. Generalized Gradient Approximation Made Simple. *Phys. Rev. Lett.* **1996**, *77*, 3865.

(4) Monkhorst, H. J.; Pack, J. D. Special Points for Brillouin-Zone Integrations. *Phys. Rev. B* **1976**, *13*, 5188.

(5) Henkelman, G.; Uberuaga, B. P.; Jónsson, H. A Climbing Image Nudged Elastic Band Method for Finding Saddle Points and Minimum Energy Paths. *J. Chem. Phys.* **2000**, *113*, 9901–9904.

(6) Liechtenstein, A. I.; Katsnelson, M.; Antropov, V.; Gubanov, V. Local Spin Density Functional Approach to the Theory of Exchange Interactions in Ferromagnetic Metals and Alloys. *J. Magn. Magn. Mater.* **1987**, *67*, 65–74.

(7) Wills, J. M.; Alouani, M.; Andersson, P.; Delin, A.; Eriksson, O.; Grechnyev, O. *Full-Potential Electronic Structure Method: Energy and Force Calculations With Density Functional and Dynamical Mean Field Theory*; Springer Science & Business Media, 2010; Vol. 167.

(8) Eriksson, O.; Bergman, A.; Bergqvist, L.; Hellsvik, J. *Atomistic Spin Dynamics: Foundations and Applications*; Oxford university press, 2017.

(9) Tersoff, J.; Hamann, D. R. Theory and Application for the Scanning Tunneling Microscope. *Phys. Rev. Lett.* **1983**, *50*, 1998–2001.

(10) Kvashnin, Y. O.; Grånäs, O.; Di Marco, I.; Katsnelson, M. I.; Lichtenstein, A. I.; Eriksson, O. Exchange Parameters of Strongly Correlated Materials: Extraction From Spin-Polarized Density Functional Theory Plus Dynamical Mean-Field Theory. *Phys. Rev. B* **2015**, *91*, 125133.

(11) Ly, T. T.; Park, J.; Kim, K.; Ahn, H.-B.; Lee, N. J.; Kim, K.; Park, T.-E.; Duvjir, G.; Lam, N. H.; Jang, K., et al. Direct Observation of Fe-Ge Ordering in $Fe_{5-x}GeTe_2$ Crystals and Resultant Helimagnetism. *Adv. Funct. Mater.* **2021**, *31*, 2009758.

(12) May, A. F.; Ovchinnikov, D.; Zheng, Q.; Hermann, R.; Calder, S.; Huang, B.; Fei, Z.; Liu, Y.; Xu, X.; McGuire, M. A. Ferromagnetism Near Room Temperature in the Cleavable van der Waals Crystal Fe_5GeTe_2. *ACS nano* **2019**, *13*, 4436–4442.

(13) Wu, X.; Lei, L.; Yin, Q.; Zhao, N.-N.; Li, M.; Wang, Z.; Liu, Q.; Song, W.; Ma, H.; Ding, P., et al. Direct Observation of Competition Between Charge Order and Itinerant Ferromagnetism in The van der Waals Crystal $Fe_{5-x}GeTe_2$. *Phys. Rev. B* **2021**, *104*, 165101.
